# Exploring the Realm of Physical Activity in Pregnant Indian Women: Understanding Their Knowledge, Practice, and Obstacles

**DOI:** 10.7759/cureus.76881

**Published:** 2025-01-03

**Authors:** Smita Singh, Mukta Agarwal, Prashant K Singh, Indira Prasad

**Affiliations:** 1 Obstetrics and Gynecology, Paras Healthcare, Patna, IND; 2 Obstetrics and Gynecology, All India Institute of Medical Sciences (AIIMS) Patna, Patna, IND; 3 Community Medicine, Netaji Subhas Medical College and Hospital, Patna, IND

**Keywords:** antenatal exercise, barrier, knowledge, motivating factors, physical activity, practice

## Abstract

Background

The study aimed to assess the knowledge of pregnant Indian women about antenatal exercises (ANEs), the challenges they face, and the motivating factors influencing their participation in physical activities. The main objective and practical implication of this research were to evaluate the prevalence of physical activity (PA) among pregnant women in Indian hospitals while also identifying the specific challenges that hinder physical activity among this demographic.

Hypothesis or aim

The hypothesis suggested that the lack of knowledge contributes to low physical activity levels, and healthcare professionals can address this through education. The research aimed to identify obstacles to physical activity among pregnant women in India.

Methods

This cross-sectional study was conducted at a tertiary healthcare facility, involving 400 pregnant women (24-36-week gestational age {GA}) receiving prenatal care. The participants completed a detailed questionnaire and interviews to assess their knowledge, routines, challenges, and sources of motivation, aiming to understand the perspectives and experiences of pregnant women.

Findings

Findings revealed that 36% of the participants had good knowledge, while 64% had poor knowledge. Only 16.5% engaged in regular exercise, with most not prioritizing physical activity. Few exercisers met the recommended daily exercise duration, with walking being the most popular activity.

Discussion

Pregnant Indian women have poor knowledge and practice of antenatal exercise compared to their Western counterparts. By addressing barriers and involving healthcare professionals, positive changes can be made to enhance understanding in this area.

Conclusion

This investigation highlights a significant gap in knowledge and practice of antenatal exercise among the participants.

## Introduction

To mitigate the risks associated with excessive weight gain and gestational diabetes mellitus (GDM), the Centers for Disease Control and Prevention (CDC) recommends that healthy pregnant women partake in a minimum of 150 minutes (30 minutes five days a week) of moderate-intensity exercise per week. This guidance is further supported by the joint CDC-American College of Sports Medicine (ACSM) recommendations, which define moderate-intensity physical activity (PA) as any exercise requiring 3-5 metabolic equivalents (METS) or activities such as brisk walking at a pace of 3-4 miles per hour or any other physical activity of similar intensity to brisk walking [1,2].

Extensive research in the realm of healthy pregnancies has identified a range of safe moderate-intensity physical activities that pregnant women can engage in, including brisk walking, jogging, stationary cycling, aerobic exercises, dancing, resistance exercises using weights or elastic bands, stretching exercises, yoga, swimming, and pelvic floor exercises. These activities not only promote physical well-being but also contribute to overall maternal health during pregnancy [2-8].

The issue of physical inactivity during pregnancy is a significant concern, as it can lead to increased maternal and fetal morbidity both before and after childbirth. In India, where only 10.7% of pregnant women adhere to the recommended CDC exercise guidelines, addressing this issue is crucial [5]. This study represents a novel contribution to the field, as prior research on antenatal exercise (ANE) knowledge, practice, and barriers among pregnant Indian women had not been conducted in India. The primary objective of the study was to evaluate the participants' understanding of the benefits of physical activity and exercise during pregnancy, as well as the perceived obstacles that may prevent them from engaging in such activities.

## Materials and methods

Research approach

This cross-sectional study was conducted in an institutional setting after approval (All India Institute of Medical Sciences {AIIMS} Patna Ethics Committee, approval number AIIMS/Pat/IEC/2022/988 and approval date 2 December 2022). The data collection involved administering an interview-based questionnaire, which was developed based on existing literature.

Study participants

Eligibility Criteria

The research involved 400 pregnant women in good health, with gestational age (GA) between 24 and 36 weeks, who attended the outpatient department from 2 December 2022 to 1 May 2023. These participants were visiting the antenatal clinic and indicated their consent to participate in the study.

Exclusion Criteria

Exclusion criteria included pregnant women who chose not to participate in the questionnaire, as well as those with medical or obstetric contraindications to exercise, as outlined by the American College of Obstetricians and Gynecologists (ACOG) guidelines and the Asia-Pacific Consensus 2021 [3,6]. Specific contraindications encompassed conditions such as placenta previa, premature labor, incompetent cervical os, persistent bleeding, uncontrolled hypertension, significant cardiovascular, respiratory or systemic disorders, multiple pregnancies, intrauterine growth restriction, uncontrolled type 1 diabetes, seizure disorders, and thyroid disorders.

Study setting

The study was carried out at a tertiary healthcare facility that caters to a population of 104.2 million individuals, with 49.8 million of them being women.

Study procedure

Eligible pregnant women were provided with a patient information sheet and then given an explanation about the purpose of the study before signing a written informed consent form. Subsequently, a qualified medical professional conducted interviews using a predetermined questionnaire.

Sample size calculation

According to the study conducted by Al-Youbi and Elsaid in 2020, the sample size calculation was performed using the OpenEpi software [9]. To estimate the expected proportion with 5% precision and 95% confidence, a sample size of 400 individuals is needed, assuming 58% have low knowledge of physical exercise.

Data collection methods

The study tool utilized in this research was developed based on a comprehensive examination of existing literature, ensuring a strong foundation for data collection. To ensure the accuracy and relevance of the questionnaire, both face and content validity checks were conducted. Additionally, to cater to the native language of the participants, the questionnaire was translated into Hindi. To further validate the linguistic aspects, the back-translation method was employed, ensuring the questionnaire's linguistic accuracy and consistency.

List of variables

The independent variables included age, gestational age (GA), body mass index (BMI), education, employment status, religion, place of residence, and family structure. The outcome variables comprised the level of knowledge and the practice of physical activity (PA), as well as the barriers and facilitators associated with PA.

Study tool

Extensive research and input from experts in the field were utilized to meticulously develop the initial questionnaire. A diverse panel of 20 experts then carefully evaluated the questionnaire to ensure its validity and reliability, assessing indexes such as item-level content validity index (I-CVI), scale-level content validity index (S-CVI), and content validity ratio (CVR). The questionnaire was structured into four sections, focusing on knowledge, practice, barriers, and motivating factors, allowing for a comprehensive understanding of the subject matter.

A standardized approach was employed to evaluate pregnant women's comprehension of antenatal exercises (ANEs) using a 15-question survey. Each question had a response code of either 0 for incorrect information or 1 for accurate knowledge. The scores ranged from 0 to 15, with the median value being 8. Scores equal to or above the median were deemed sufficient, while scores below were considered insufficient.

The ANE barriers were categorized into intrapersonal, interpersonal, and environmental groups, with scores ranging from 16 to 60 across 15 questions. The median barrier score of 24 was classified as median low (<24) and median high (>24) for further analysis. After conducting a pilot study with 20 pregnant women, changes were made to the questionnaire. The participants were classified as regular exercisers (150+ minutes per week), infrequent exercisers (less than 150 minutes per week), and non-exercisers.

Content validation

Content validation consists of six distinct steps: 1) preparing the content validation form; 2) assembling a panel of expert reviewers; 3) implementing the content validation process through an online platform; 4) evaluating the domain and individual items; 5) assigning a score to each item based on the item-level content validity index (I-CVI), which reflects the percentage of content experts rating the item as relevant (3 or 4); and 6) calculating the content validity index (CVI).

Content validity ratio

The appropriateness of each question was evaluated by a panel of 20 experts using a four-point scale: nonrelevant, somewhat relevant, quite relevant, and highly relevant. The CVR for each question was then calculated using Lawsche's formula. The calculated CVRs were compared to the minimum required value of 0.44 for 20 raters, as specified in Lawsche's table [10].

Content validity index

The questionnaire's content validity was rigorously assessed using I-CVI and S-CVI, with experts rating each question on a Likert scale to determine relevance. The reliability was confirmed through a test-retest method with 20 experts, showing strong internal consistency and a high Cronbach's alpha value of 0.886 for the total score. The questionnaire also demonstrated reliability and reproducibility with a total intra-class coefficient of 0.886 and a 95% confidence interval of 0.703-0.956.

Statistical analysis

Collected data was entered in Microsoft Excel 2019 (Microsoft Corp., Redmond, WA) from the data collection instrument. The cleaning and coding of data were done in Microsoft Excel 2019, and then, the Excel sheet was imported and analyzed using Statistical Package for Social Sciences (SPSS) v.20 (IBM Corp., Armonk, NY). Knowledge and barrier scores were graphically represented through error bar diagrams. Categorical variables were described using frequency (proportions) and analyzed using the chi-square test or Fisher's exact test as applicable. Quantitative variables meeting assumptions of normality were described using mean ± standard deviation (SD) and analyzed using Student's t-test.

## Results

Participant sociodemographic and obstetric characteristics

The study included 400 pregnant women aged 15-48 years, with an average age of 25.61 years and a standard deviation of 4.96 years. In the study, 54.3% were multigravida, and 45.8% were primigravida, with an average gestational age of 29.44 weeks and a mean BMI of 25.84 ± 3.090. Fifty-four percent completed high school, and 4.55% were illiterate. Residential distribution was 44.5% rural, 16.7% semi-urban, and 38.8% urban. Ninety-two percent were homemakers, 80.5% were in joint families, and 7.7% were employed (Table 1).

**Table 1:** Sociodemographic characteristics of the study participants. BMI: body mass index

Variables	Categories	Number (n = 400)	Percentage (%)
1. Age (years)	15-19	24	6%
	20-24	168	42%
	25-29	143	35.8%
	30-34	46	11%
	35-39	13	3.2%
	40-49	8	2%
	Mean age = 25.61 ± 4.961		
2. BMI	<18.5	3	0.75%
	18.5-24.9	147	36.75%
	25-29.9	209	52.25%
	>30	41	10.25%
	Mean BMI = 25.84 ± 3.090		
3. Religion	Hindu	346	86.5%
	Muslim	51	12.8%
	Christian	3	0.7%
4. Education	Illiterate	18	4.5%
	Primary	34	8.5%
	Secondary	132	33%
	Graduate and above	216	54%
	Rural	178	44.5%
5. Residence	Semi-urban	67	16.7%
	Urban	155	38.8%
	Nuclear	78	19.5%
6. Family	Joint	322	80.5%
	Homemaker	369	92.3%
7. Occupation	Employed mostly in indoor occupation	16	4%
	Employed mostly in outdoor occupation	15	3.7%
8. Physically activity before pregnancy	Yes	26	6.5%
	No	374	93.5%

In our comprehensive analysis, we discovered that 61.2% of the participants had a prior experience with vaginal deliveries, while 38.8% had undergone lower-segment cesarean sections. An inquiry into previous abortion histories revealed that 20.8% had experienced one or more abortions, in contrast to 79.2% who had no such history. Furthermore, within the study cohort, a mere 6.5% maintained a physically active lifestyle prior to pregnancy, leaving a substantial 93.5% who were physically inactive before embarking on their journey to motherhood. The most prevalent medical risk factors identified were anemia, affecting 9% of the participants, and gestational diabetes mellitus (GDM), which impacted 3% (Table 2).

**Table 2:** Obstetric characteristics of the study participants. LSCS, lower-segment cesarean section; GDM, gestational diabetes mellitus; PIH, pregnancy-induced hypertension; IHCP, intrahepatic cholestasis of pregnancy

Obstetric characteristics		Number	Percentage
Parity	Primigravida	183	45.8%
	Multigravida	217	54.3%
Gestational age (GA) (weeks)	24-27	179	44.75%
	29-31	70	17.5%
	32-36	151	37.75%
	Mean GA = 29.44 ± 4.415		
Previous mode of delivery	Vaginal	123	61.2%
	LSCS	78	38.8%
Previous history of abortion	Yes	83	20.8%
	No	317	79.2%
Medical risk	Hypothyroidism	29	7.25%
	GDM	12	3%
	PIH	9	2.25%
	Mild anemia	36	9%
	IHCP	5	1.25%

Knowledge assessment and its association

Out of the 400 pregnant mothers surveyed, it was found that 220 of them, or 55%, had heard about prenatal exercise. Among those who had heard about it, 216 (54%) and 205 (51.2%) acknowledged that they knew it was beneficial for both the mother and fetus and that it helped prevent excessive weight gain and obesity. However, a significant number of participants lacked knowledge in certain areas. For instance, 314 (78.5%) were unaware of safe exercises during pregnancy, and 310 (77.6%) did not know the recommended duration of exercise for a healthy pregnancy. On the other hand, 169 (42.3%) were aware that regular physical activity reduces the risk of gestational diabetes mellitus (GDM) and hypertension, while 178 (44.5%) knew that it aids in achieving a normal vaginal birth. Interestingly, the question regarding the knowledge of exercise lowering the risk of postnatal depression received the fewest responses, with only 19.3% of the participants being aware of this (Table 3).

**Table 3:** Knowledge questionnaire. PA, physical activity; GDM, gestational diabetes mellitus

Serial number	Questionnaire	No (code 0)	Do not know (code 0)	Yes (code 1)
		n (%)	n (%)	n (%)
1	Have you ever heard about antenatal exercises (ANEs)?	91 (22.8%)	89 (22.2%)	220 (55%)
2	Do you know that ANEs are beneficial for the mother and fetus?	41 (10.3%)	143 (35.8%)	216 (54%)
3	Do you know the recommended duration of ANE in a healthy pregnancy?	95 (23.8%)	215 (53.8%)	90 (22.5%)
4	Do you know which exercises are safe for pregnant women?	124 (31%)	190 (47.5%)	86 (21.5%)
5	Do you know that only household activities are not sufficient and you need to do recommended exercises for both physical and mental fitness?	54 (13.5%)	170 (42.5%)	176 (44%)
6	Do you know that ANE prevents excessive weight gain and obesity?	43 (10.8%)	152 (38%)	205 (51.2%)
7	Do you know that regular PA lowers the chances of GDM and hypertension?	73 (18.3%)	158 (39.5%)	169 (42.3%)
8	Do you know that regular PA facilitates normal vaginal delivery?	60 (15%)	162 (40.5%)	178 (44.5%)
9	Do you know that ANE reduces the risk of perinatal and postnatal back pain?	64 (16%)	226 (56.5%)	110 (27.5%)
10	Do you know that ANE enhances energy and stamina?	55 (13.8%)	155 (38.8%)	190 (47.5%)
11	Do you know that ANE reduces lower limb edema?	123 (30.8%)	134 (33.5%)	143 (35.8%)
12	Do you know that ANE reduces the risk of postnatal depression?	113 (28.2%)	210 (52.5%)	77 (19.3%)
13	Do you know that ANE strengthens the pelvic floor muscle?	80 (20%)	186 (46.5%)	134 (33.5%)
14	Do you know the conditions in which ANEs are not safe?	126 (31.5%)	154 (38.5%)	120 (30%)
15	Do you know that exercise in pregnancy gives more rapid postnatal recovery?	113 (28.2%)	170 (42.5%)	117 (29.3%)

Overall, the study found that 144 (36%) had an adequate knowledge score, while 256 (64%) had inadequate knowledge. Surprisingly, no significant correlation was found between the participants' sociodemographic or obstetric characteristics and knowledge adequacy (Table 4).

**Table 4:** Association of knowledge adequacy with different characteristics. A p-value equal to or less than 0.05 was considered "significant." A p-value of >0.05 was considered "not significant." LSCS: lower-segment cesarean section

Serial number	Characteristics	Adequacy of knowledge		Chi-square (χ^2^) test	P-value
		Inadequate knowledge (less than median score of 8)	Adequate knowledge (more than or equal to median score of 8)		
		N (%)	N (%)		
1	Age (years)				
	15-34 years	242 (60.5%)	138 (34.5%)	0.329	0.566
	35-50 years	14 (3.5%)	6 (1.5%)		
2	Parity				
	Primigravida	115 (28.7%)	68 (17%)	0.196	0.658
	Multigravida	141 (35.3%)	76 (19%)		
3	Religion				
	Hindu	220 (55%)	126 (31.5%)	0.193	0.661
	Other than Hindu	36 (9%)	18 (4.5%)		
4	Education				
	Primary and illiterate	38 (9.5%)	14 (3.5%)	2.718	0.257
	Secondary	86 (21.5%)	46 (11.5%)		
	Graduate and above	132 (33%)	84 (21%)		
5	Residence				
	Rural	113 (28.2%)	65 (16.3%)	0.039	0.98
	Semi-urban	43 (10.8%)	24 (6%)		
	Urban	100 (25%)	55 (13.8%)		
6	Family				
	Nuclear	46 (11.5%)	32 (8%)	1.062	0.303
	Joint	210 (52.5%)	112 (28%)		
7	Occupation				
	Homemaker	240 (60%)	129 (32.3%)	3.125	0.21
	Employed mostly in indoor occupation	7 (1.8%)	7 (1.8%)		
	Employed mostly in outdoor occupation	9 (2.3%)	9 (2.3%)		
8	Previous mode of delivery				
	Vaginal	82 (40.8%)	41 (20.4%)	0.035	0.851
	LSCS	51 (25.4%)	27 (13.4%)		
9	Previous history of abortion				
	No	205 (51.2%)	112 (28%)	0.297	0.586
	Yes	51 (12.8%)	32 (8%)		
10	Physical activity (PA) before pregnancy				
	No	237 (59.8%)	133 (33.6%)	0.425	0.515
	Yes	15 (3.8%)	11 (2.8%)		
11	Medical risk				
	No	193 (52.7%)	116 (31.7%)	0.366	0.545
	Yes	38 (10.4%)	19 (5.2%)		

However, the study did reveal some interesting relationships between sociodemographic and obstetric variables and the practice of prenatal exercise (Table 5).

**Table 5:** Association of ANE practice with different characteristics. A p-value equal to or less than 0.05 was considered "significant." A p-value of >0.05 was considered "not significant." ANE, antenatal exercise; LSCS, lower-segment cesarean section

Serial number	Characteristics	Was ANE practiced?		Chi-square (χ^2^) test	P-value
		No	Yes		
		n (%)	n (%)		
1	Age (years)				
	15-34 years	336 (84%)	44 (11%)	1.276	0.259
	35-50 years	16 (4%)	4 (1%)		
2	Parity				
	Primigravida	169 (42.3%)	14 (3.5%)	6.044	0.015
	Multigravida	183 (45.8%)	34 (8.5%)		
3	Religion				
	Hindu	303 (75.8%)	43 (10.8%)	0.444	0.505
	Other than Hindu	49 (12.3%)	5 (1.3%)		
4	Education				
	Primary and illiterate	47 (11.8%)	5 (1.3%)	0.939	0.625
	Secondary	118 (29.5%)	14 (3.5%)		
	Graduate and above	187 (46.8%)	29 (7.2%)		
5	Residence				
	Rural	158 (39.5%)	20 (5%)	0.218	0.897
	Semi-urban	59 (14.8%)	8 (2%)		
	Urban	135 (33.8%)	20 (5%)		
6	Family				
	Nuclear	66 (16.5%)	12 (3%)	1.051	0.305
	Joint	286 (71.5%)	36 (9%)		
7	Occupation				
	Homemaker	330 (82.5%)	39 (9.8%)	9.386	0.011
	Employed mostly in indoor occupation	11 (2.8%)	5 (1.3%)		
	Employed mostly in outdoor occupation	11 (2.8%)	4 (1%)		
8	Previous mode of delivery				
	Vaginal	106 (52.7%)	17 (8.5%)	2.35	0.125
	LSCS	64 (31.8%)	14 (7%)		
9	Previous history of abortion				
	No	283 (70.8%)	34 (8.5%)	0.297	0.586
	Yes	69 (17.3%)	14 (3.5%)		
10	Physical activity before pregnancy				
	No	329 (83.1%)	41 (10.4%)	5.724	0.017
	Yes	19 (4.8%)	7 (1.8%)		
11	Medical risk				
	No	270 (73.8%)	39 (10.7%)	0.086	0.769
	Yes	49 (13.4%)	8 (2.2%)		
12	Was ANE advised by anyone?				
	No	320 (80%)	14 (3.5%)	116.875	0.00
	Yes	32 (8%)	34 (8.5%)		

**Figure 1:**
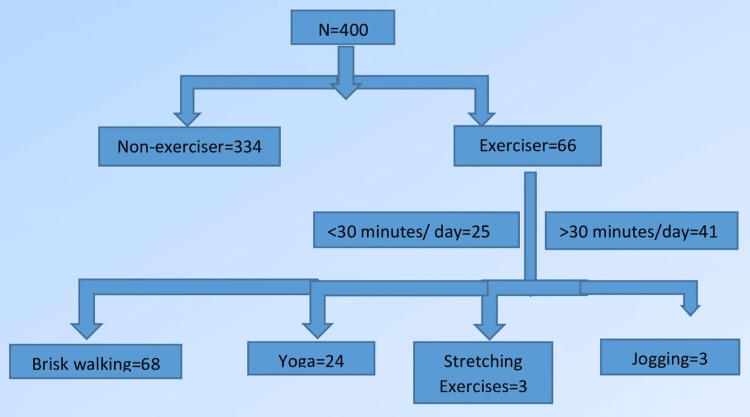
Flow diagram showing antenatal exercise (ANE) practice.

It was observed that multigravida and homemakers were more likely to engage in prenatal exercise. Additionally, those who exercised before becoming pregnant were also more likely to continue exercising during pregnancy (p-value = 0.017). Although no significant association was found between the practice of prenatal exercise and barriers, it is worth noting that out of the 400 participants, 334 (83.5%) were non-exercisers and only 66 (16.5%) were exercisers. Furthermore, only 41 participants (10.2%) in the exerciser group met the minimum recommendation of exercising for at least 30 minutes each day. The most popular forms of exercise among the exercisers were brisk walking and yoga (Figure 1).

Association of barrier scores

**Figure 2:**
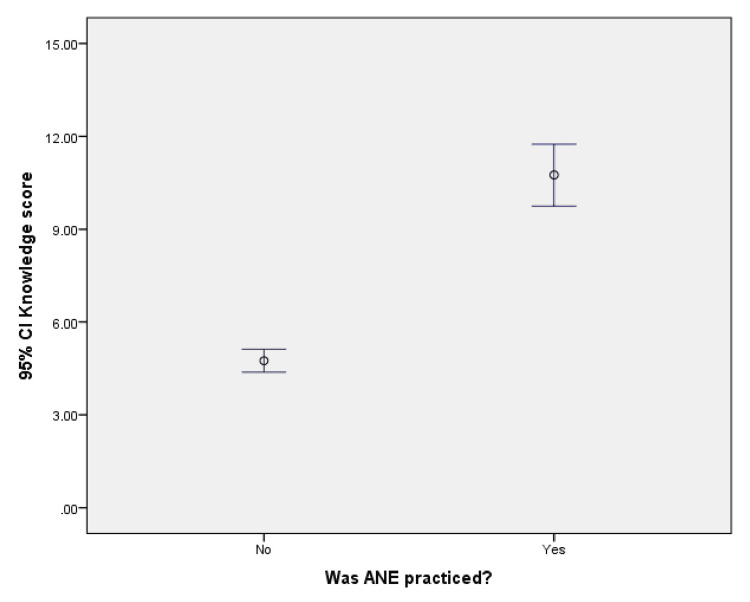
Error bar diagram showing the correlation between knowledge score and exercise practice. CI, confidence interval; ANE, antenatal exercise

**Figure 3:**
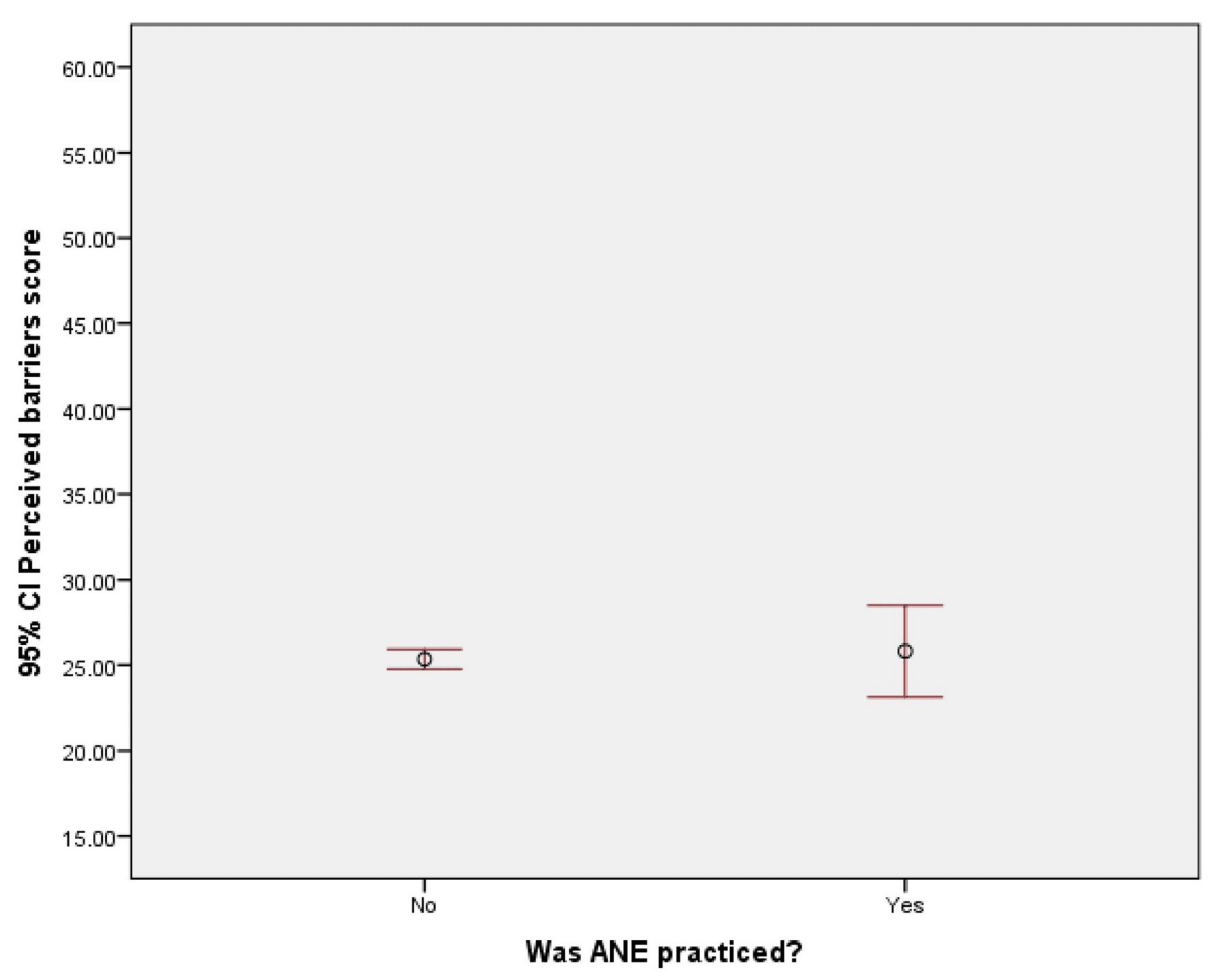
Error bar diagram showing that the perceived barrier score of ANE was not associated with its practice. CI, confidence interval; ANE, antenatal exercise

An analysis using error bar diagrams was conducted to examine the relationship between exercise practice, knowledge scores, and perceived barrier scores among pregnant women. The findings revealed that pregnant women with higher knowledge scores tended to engage in more aerobic and anaerobic exercise (ANE), while perceived barriers did not have a significant impact on exercise practice. These results are illustrated in Figure 2 and Figure 3.

Furthermore, the study also found that there was no significant correlation between perceived barrier scores and other characteristics among the participants, as shown in Table 6. This suggests that perceived barriers did not influence factors such as exercise practice, knowledge scores, or other traits in pregnant women.

**Table 6:** Association of barrier scores with different characteristics. A p-value equal to or less than 0.05 was considered "significant." A p-value of >0.05 was considered "not significant." LSCS, lower-segment cesarean section; SD, standard deviation

Serial number	Characteristics	Barrier score		P-value
		Median low (<24)	Median high (>24)	
1	Age (years) (mean ± SD)	25.89 ± 5.12	25.42 ± 4.84	0.354
2	Parity			
	Primigravida	79	104	0.543
	Multigravida	87	130	
3	Religion			
	Hindu	147	199	0.373
	Other than Hindu	19	35	
4	Education			
	Primary and illiterate	21	31	0.193
	Secondary	63	69	
	Graduate and above	21	134	
5	Residence			
	Rural	80	98	0.398
	Semi-urban	24	43	
	Urban	62	93	
6	Family			
	Nuclear	35	43	0.524
	Joint	131	191	
7	Occupation			
	Homemaker	156	213	0.483
	Employed mostly in indoor occupation	6	10	
	Employed mostly in outdoor occupation	4	11	
8	Previous mode of delivery			
	Vaginal	52	71	0.302
	LSCS	27	51	
9	Previous history of abortion			
	No	130	187	
	Yes	36	47	
10	Physical activity before pregnancy			
	No	149	221	0.100
	Yes	15	11	
11	Medical risk			
	No	129	180	0.347
	Yes	20	37	

The survey results indicated that the majority of pregnant women, with percentages ranging from 89.3% to 98.8%, believed that various forms of support and encouragement would motivate them to engage in exercise. Seeking advice from a medical professional, spouse's support, encouragement from family and friends, participation in clinic-based fitness programs and community-based fitness programs, and media advertisements were all identified as potential motivators for exercise. These findings are detailed in Table 7, highlighting the importance of social support and external influences in promoting physical activity among pregnant women.

**Table 7:** Motivating factors of the study participants.

Motivating factors		n	Percentage (%)
Motivation by healthcare professional advice	No	5	1.30%
	Yes	395	98.8%
Motivation by partner support	No	12	3.0%
	Yes	388	97.0%
Motivation by family and friends	No	34	8.5%
	Yes	366	91.5%
Motivation by exercise sessions at clinics	No	28	7.0%
	Yes	372	93.0%
Motivation by community-based exercise program	No	25	6.3%
	Yes	375	93.8%
Motivation by promotional message in mass media	No	43	10.8%
	Yes	357	89.3%

## Discussion

Engaging in regular exercise is an essential aspect of maintaining a healthy lifestyle, even throughout the duration of pregnancy. Obstetrician-gynecologists and other healthcare providers specializing in obstetric care strongly advocate for the inclusion of exercise in a pregnant woman's routine. Exercise is defined as a purposeful and structured physical activity that involves repetitive bodily movements aimed at enhancing various aspects of physical fitness. The American College of Obstetricians and Gynecologists (ACOG) has provided clear recommendations that highlight the positive impact of physical activity during pregnancy. These recommendations emphasize that engaging in exercise while pregnant can increase the likelihood of vaginal delivery and reduce the occurrence of excessive weight gain, gestational diabetes mellitus (GDM), gestational hypertensive disorders, preterm birth, cesarean section rates, operative vaginal delivery rates, low birth weight, and postpartum depression [6].

In the current study conducted in Bihar, Eastern India, a total of 400 pregnant Indian women participated. Bihar, according to the most recent census, has a population of 104,099,452 people, with 54,278,157 men and 49,821,295 women. The study revealed that only a very small percentage of pregnant women met the requirements for physical activity (PA), indicating that the majority of pregnant women in Bihar are leading sedentary lifestyles. Out of the 400 participants, 334 (83.5%) were non-exercisers, while 66 (16.5%) were exercisers. Surprisingly, only 41 individuals (10.2%) in the exerciser group managed to meet the daily exercise recommendation of at least 30 minutes. The global examination of physical activity during pregnancy by Silva-Jose et al. found that 59.09% of the studies showed low levels of physical activity [7]. Similarly, a study conducted in Saudi Arabia by Alaglan et al. discovered that 58% of pregnant women in their sample did not exercise [8]. Another cross-sectional study in Saudi Arabia by Al Youbi and Elsaid et al. found that 84.2% of pregnant women did not engage in exercise, primarily due to fatigue. Interestingly, they also observed that 50.67% of the participants had a good understanding of physical activity, which aligns with our study's finding that 64% had an appropriate level of knowledge. However, they did find a statistically significant difference in educational attainment and work position between pregnant women with high and low knowledge levels, but no significant association was found between practice and knowledge levels [9].

The results of our study revealed that brisk walking emerged as the most favored choice of exercise among the participants, with a majority of 36 out of 66 individuals opting for it. Following closely behind was yoga, which was chosen by 24 out of 66 individuals. Interestingly, only a small percentage (9.5%) of women received advice to avoid exercise due to medical or obstetric complications. In contrast, we found a significant association between being a homemaker and engaging in exercise during pregnancy, as opposed to women who were employed. This could be attributed to the belief among working women that they already engage in sufficient physical activity through their jobs.

Evenson et al.'s Pregnancy, Infection, and Nutrition (PIN) Study in North Carolina surveyed 1,535 pregnant women in their 27-30 weeks of pregnancy to identify obstacles to physical activity. Results showed that 85% cited personal barriers such as insufficient healthcare advice [11]. Flannery et al. discovered that the lack of knowledge was the main barrier to physical activity for pregnant women, emphasizing the need to consider diverse populations and contexts in studying this issue [12]. A recent study in Iran revealed that the lack of family support is a major obstacle to physical activity for pregnant women, emphasizing the importance of involving family members in interventions [13]. Alrzeghi and Elbsheni's study with 200 pregnant women found that the difficulty of exercising during pregnancy was the most common barrier, highlighting the need for tailored exercise programs to address these physical challenges [14].

Our research found that the main obstacles are the absence of proper healthcare guidance and limited knowledge. A significant barrier for 47.5% of expectant mothers was identified as the absence of healthcare advice, whereas 18% pointed to a deficiency in knowledge. This highlights the urgent need for improved access to healthcare information and education for pregnant women.

In 2020, a comprehensive literature review conducted by a group of 18 experts, including a gynecologist, resulted in the Asia-Pacific Consensus providing seven essential recommendations for physical activity during pregnancy and the postpartum phase [3]. The primary objective of these recommendations was to enhance the metabolic health of pregnant women in the Asia-Pacific region by offering guidance and advice to healthcare professionals. Additionally, a systematic literature review conducted by Harrison et al. encompassing 47 studies revealed that personal barriers such as fatigue, the lack of time, and pregnancy discomforts were the most prevalent obstacles to physical activity during pregnancy [15]. On the other hand, factors such as feto-maternal health benefits, social support, and pregnancy-specific programs were identified as significant facilitators of physical activity during pregnancy. Furthermore, a scoping literature review conducted by Okafor and Goon, which included 13 research articles, highlighted the importance of exercise advice or counselling provided by healthcare providers in promoting adherence to physical activity during pregnancy and the postnatal period [16]. This finding aligns with our own study, where an overwhelming 98.8% of the participants expressed their belief that seeking advice from a medical professional would motivate them to engage in exercise.

Strength and limitations

The study had a strong methodology, including a large sample size and a comprehensive measurement of factors such as knowledge, practice, and barriers during pregnancy. However, it only focused on low-risk pregnancies, limiting its applicability. The study did not establish a clear link between practice and perceived barriers, which is a significant shortcoming. Future research should consider qualitative and longitudinal approaches to better understand these factors during pregnancy.

## Conclusions

Education and understanding about the importance of exercise, particularly during pregnancy, are crucial in today's world where chronic medical conditions related to obesity are on the rise among women. The risk of postpartum weight gain is a significant concern, especially in developing countries where sedentary lifestyles are prevalent. Our research shows that pregnant women lack knowledge about the benefits of prenatal exercise, leading to low levels of physical activity during pregnancy. This can have negative health effects for both mother and baby. However, we found that well-informed pregnant women are more likely to exercise regularly. It is important to provide accurate information and support to expectant mothers to promote a healthy lifestyle during pregnancy. Healthcare providers are crucial in educating pregnant women about the benefits of exercise and helping them overcome obstacles to staying active during pregnancy.

## References

[1] (2015). ACOG committee opinion no. 650: physical activity and exercise during pregnancy and the postpartum period. Obstet Gynecol.

[2] (2023). Physical activity recommendations for pregnant and postpartum women. https://www.cdc.gov/physicalactivity/basics/pregnant-and-postpartum-women.html.

[3] Lee R, Thain S, Tan LK, Teo T, Tan KH (2021). Asia-Pacific consensus on physical activity and exercise in pregnancy and the postpartum period. BMJ Open Sport Exerc Med.

[4] Rudin LR, Dunn L, Lyons K, Livingston J, Waring ME, Pescatello LS (2021). Professional exercise recommendations for healthy women who are pregnant: a systematic review. Womens Health Rep (New Rochelle).

[5] Anjana RM, Sudha V, Lakshmipriya N (2016). Physical activity patterns and gestational diabetes outcomes - the wings project. Diabetes Res Clin Pract.

[6] (2020). Physical activity and exercise during pregnancy and the postpartum period. Obstet Gynecol.

[7] Silva-Jose C, Sánchez-Polán M, Barakat R, Gil-Ares J, Refoyo I (2022). Level of physical activity in pregnant populations from different geographic regions: a systematic review. J Clin Med.

[8] Alaglan AA, Almousa RF, Alomirini AA (2020). Saudi women’s physical activity habits during pregnancy. Womens Health (Lond).

[9] Al-Youbi GM, Elsaid T (2020). Knowledge, attitude, and practices on exercise among pregnant females attending Al-Wazarat Health Center, Riyadh, Saudi Arabia. J Family Med Prim Care.

[10] Wilson FR, Pan W, Schumsky DA (2012). Recalculation of the critical values for Lawshe’s content validity ratio. Meas Eval Couns Dev.

[11] Evenson KR, Moos MK, Carrier K, Siega-Riz AM (2009). Perceived barriers to physical activity among pregnant women. Matern Child Health J.

[12] Flannery C, McHugh S, Anaba AE (2018). Enablers and barriers to physical activity in overweight and obese pregnant women: an analysis informed by the theoretical domains framework and COM-B model. BMC Pregnancy Childbirth.

[13] Ahmadi K, Amiri-Farahani L, Haghani S, Hasanpoor-Azghady SB, Pezaro S (2021). Exploring the intensity, barriers and correlates of physical activity In Iranian pregnant women: a cross-sectional study. BMJ Open Sport Exerc Med.

[14] Alrzeghi N, Elbsheni F (2021). Perceived barriers to physical activity during pregnancy. J Womens Health Issues Care.

[15] Harrison AL, Taylor NF, Shields N, Frawley HC (2018). Attitudes, barriers and enablers to physical activity in pregnant women: a systematic review. J Physiother.

[16] Okafor UB, Goon DT (2021). Physical activity advice and counselling by healthcare providers: a scoping review. Healthcare (Basel).

